# Evaluation of IScore validity in a Greek cohort of patients with type 2 diabetes

**DOI:** 10.1186/1471-2377-13-121

**Published:** 2013-09-16

**Authors:** Vasileios Dragoumanos, Konstantinos N Tzirogiannis, Georgios I Panoutsopoulos, Konstantinos Krikonis, Evangelos Fousteris, Maria Vourvou, Georgios Elesnitsalis, Nikolaos Melas, Kalliopi T Kourentzi, Andreas Melidonis

**Affiliations:** 1 Diabetes Center, “Tzanio” General Hospital of Piraeus, Piraeus, Greece; 2 Department of Nursing, Faculty of Human Movement and Quality of Life Science, University of Peloponnese, Sparta, Lakonia, Greece; 3 Mathematics Department, Aristotle University of Thessaloniki, Thessaloniki, Greece; 4 Intensive Care Unit, “Papageorgiou” General Hospital of Thessaloniki, Thessaloniki, Greece; 5 1st Internal Medicine Department, “Tzanio” General Hospital of Piraeus, Piraeus, Greece; 6 Medical School, University of Ioannina, Ioannina, Greece

**Keywords:** IScore, Stroke, Ischemic stroke, Ischemic stroke mortality, Diabetes

## Abstract

**Background:**

Diabetes constitutes a risk factor for stroke that also aggravates stroke prognosis. Several prognostic models have been developed for the evaluation of neurologic status, severity, short-term functional outcome and mortality of stroke patients. IScore is a novel tool recently developed in order to predict mortality rates within 30 days and 1 year after ischemic stroke and diabetes is not included in the scoring scale of IScore. The aim of the present study was to evaluate and compare IScore validity in ischemic stroke patients with and without diabetes.

**Methods:**

This prospective study included 312 consecutive Caucasian patients with type 2 diabetes and 222 Caucasian patients without diabetes admitted for ischemic stroke in a tertiary Greek hospital. Thirty-day and 1-year IScores were individually calculated for each patient and actual mortality was monitored at the same time intervals. IScore’s predictive ability and calibration was evaluated and compared for ischemic stroke patients with and without diabetes. The performance of IScore for predicting 30 and 1-year mortality between patients with and without diabetes was assessed by determining the calibration and discrimination of the score. The area under the receiver operating characteristic curve was used to evaluate the discriminative ability of IScore for patients with and without diabetes, whereas the calibration of IScore was assessed by the Hosmer–Lemeshow goodness-of fit statistic.

**Results:**

Baseline population characteristics and mortality rates did not differ significantly for both cohorts. IScore values were significantly higher for patients with diabetes at 30 days and 1 year after ischemic stroke and patients with diabetes presented more frequently with lacunar strokes. Based on ROC curves analysis IScore’s predictive ability for 30 day mortality was excellent, without statistically significant difference, for both cohorts. Predictive ability for 1 year mortality was also excellent for both groups with significantly better ability for patients with diabetes especially at high score values. Calibration of the model was good for both groups of patients.

**Conclusions:**

IScore accurately predicts mortality in acute ischemic stroke Caucasian patients with and without diabetes with higher efficacy in predicting 1 year mortality in patients with diabetes especially with high scores.

## Background

Stroke is a leading cause of adult morbidity and mortality worldwide [1,2] and diabetes constitutes a major risk factor for stroke [3-7]. Diabetes not only confers an increased risk for stroke but is also connected with increased stroke severity and mortality and poorer post-stroke functional recovery [4,8-10]. In this context accurate evaluation of early stroke prognosis in patients with diabetes, as well as in stroke patients in general, is fundamental for guiding interventions and establishing an evidence based health care decision system.

IScore is a recently developed prognostic model by Canadian Researchers for the prediction of 30-day and 1-year mortality after ischemic stroke (IS) that has not been evaluated outside the Canadian Health Care System [11,12]. Diagnosis of diabetes is not included in IScore prediction model where only hyperglycemia upon admission is taken into account. The aim of the present study was to evaluate IScore validity in IS patients with diabetes and to compare its efficacy with that in patients without diabetes.

## Methods

### Patients and methods

This prospective study was conducted in a tertiary Greek hospital. The study population consisted of 312 consecutive Caucasian patients with type 2 diabetes and 222 Caucasian non-diabetic patients admitted between January 2008 and February 2011 for acute IS. Acute stroke was defined according to the World Health Organization criteria [13] and clinical diagnosis was confirmed by brain computed tomography (CT). Categorization of IS subtypes was made according to the Trial of ORG 10172 in Acute Stroke Treatment (TOAST) [14]. Comatose patients, patients with hemorrhagic stroke, tumors, or other conditions mimicking at presentation thrombotic stroke or transient ischemic attacks were excluded from the study. The study was approved by the Tzanion General Hospital Scientific Board and Ethics Committee and informed consent was obtained on all cases.

Thirty-day and 1-year scores were calculated for each patient and actual mortality was monitored at the same time intervals. Thirty-day and 1-year scores were calculated independently and blinded to mortality data for each patient by two neurology specialists (Table 1). Occurrence of actual mortality was examined by telephone from a third specialist blinded to IScore data. Missing or patients that did not respond were categorized as lost to follow up.

**Table 1:** Demographic baseline population characteristics and IScore variables at 30-days and 1-year

**Variable**	**Entire population**	**Diabetic patients (n=312)**	**Non diabetic patients (n=222)**	**p-value**
**iScore 30 days**	166,1 ± 56,3	185,3 ± 45,9	139,1 ± 58,7	<0,05^#^
**iScore 1 year**	136,3 ± 41	149,1 ± 22,6	118,3 ± 43,6	<0,05^#^
**Age (mean±SD)**	74,7 ± 6,6	75.03 ± 6.6	74.22 ± 6.5	0,222^##^
		74 (69 - 82)	74 (69 - 80)	
**Gender n (%)**				0,628*
Female	276	158 (50.6)	118 (53.2)	
Male	258	154 (49.4)	104 (46.8)	
**Stroke severity (CNS) n (%)**				0,247*
0	21	13 (4.2)	7 (3.2)	
≤4	75	36 (11.5)	39 (17.6)	
5-7	204	123 (39.4)	81 (36.5)	
≥8	235	140 (44.9)	95 (42.8)	
**Stroke subtype n (%)**				<0,05*
Lacunar origin	244	168 (53.8)	76 (34.2)	
Nonlacunar origin	219	102 (32.7)	117 (52.7)	
Undetermined origin	71	42 (13.5)	29 (13.1)	
**Risk factor n (%)**				0,304*
Atrial fibrillation	80	46 (14.7)	34 (15.3)	
CHF	67	44 (14.1)	23 (10.4)	
Previous MI	148	99 (31.7)	49 (22.1)	
Current smoker	100	57 (18.3)	43 (19.4)	
**Comorbid condition n (%)**				0,809**
Cancer	53	34 (10.9)	19 (8.6)	
Renal dialysis	9	5 (1.6)	4 (1.8)	
**Preadmission disability n (%)**				0,532*
Independent	435	254 (81.4)	181 (81.5)	
Dependent n	99	58 (18.6)	41 (18.5)	
**Glucose on admission, mmol/L,dL n (%)**				<0,05*
<7.5 (<135)	295	159 (51)	136 (61.3)	
≥7.5 (≥135)	239	153 (49)	86 (38.7)	
**Mortality rate n (%)**				0,551*
30 Day	77	45 (14.4)	32 (14.4)	
1 Year	141	86 (27.6)	55 (24.8)	0,268*

SD indicates standard deviation; CNS: Canadian neurological scale; CHF: congestive heart failure; MI: myocardial infarction.

Statistical methods used: ^#^Mann Whitney U test, ^##^t-Test, ^*^Chi-square, ^**^Fisher.

IScore's mortality predictors (according to original publication) included older age, male sex, stroke severity, non-lacunar stroke subtype, glucose ≥ 7.5 mmol/L (135 mg/dL) upon admission, history of atrial fibrillation, coronary artery disease (CAD), congestive heart failure (CHF), cancer, dementia, kidney disease on dialysis and dependency prior to stroke [11].

### Statistical analysis

Numerical variables were presented as mean ± standard deviation, while discrete variables as absolute values and summarized by percentages. Categorical variables were analyzed using chi-square test or Fisher’s exact test. To compare mean or, when appropriate, median differences for continuous variables in baseline characteristics between groups Student’s *t*-Test or Mann–Whitney *U* test were used. The performance of IScore for predicting 30-days and 1-year mortality between diabetic and non-diabetic patients was assessed by determining the calibration and discrimination of the score.

The area under the receiver operating characteristic curve was used to evaluate the discriminative ability of IScore for patients with and without diabetes. The area under the curve (AUC) [15] was calculated as an index of how well IScore could discriminate patients who lived and those who died both in 30-day and 1-year after admission. The discriminative power of the model was considered excellent if the area under the receiver operating characteristic curve was >0.80, very good if >0.75 and good if >0.70 [16].

The calibration of IScore was assessed by the Hosmer–Lemeshow goodness-of fit statistic [17]. For the Hosmer–Lemeshow statistic, the predicted risks of individual patients were rank-ordered and divided into 8 risk categories based on quintiles according to initial IScore publication [11]. Within each group of estimated risk, the number of predicted deaths was accumulated against the number of observed deaths and p > 0.05 was considered to indicate acceptable calibration of the model. Baseline characteristic analysis was performed using the IBM SPSS for Windows v.20 software (IBM, New York, USA) and ROC curve analysis was completed with STATAv12 (StataCorp LP, Texas, USA).

## Results

Demographic baseline population characteristics (Table 1) did not differ significantly between IS patients with diabetes and patients without diabetes. Forty-five (14.4%, 95% CI, 0.11-0.18) patients with diabetes deceased 30 days after IS and 86 (27.6%, 95% CI, 0.23-0.33) after 1 year and mortality rates were similar for non-diabetic patients: 32 (14.4%, 95% CI, 0.1-0.19) at 30 days and 55 (24.8%, 95% CI, 0.22-0.34) at 1 year. Mean IScore values at 30 days and 1 year were 185.3 ± 45.9 and 149.1 ± 22.1 respectively for patients with diabetes and 139.1 ± 58.7 and 118.3 ± 43.6 for non-diabetic patients and the above values were significantly higher in patients with diabetes (Table 1). Regarding stroke subtypes a statistically significant difference was observed between groups with preponderance of lacunar strokes in IS patients with diabetes (Table 1). Demographic population characteristics and IScore variables are also presented after age stratification (<65, 66-80 and >80 years old) in Table 2.

**Table 2:** Demographic baseline population characteristics and IScore variables at 30-days and 1-year for different age groups (<65, 66-80 and >80 years old)

**Age categories**		**<65**	**66-80**	**>80**
		**Mean ± SD**	**Mean ± SD**	**Mean ± SD**
**Age (years)**		63,7 ± 1,2	72,3 ± 3,8	83,2 ± 2,3
**iScore 30 days**		120,6 ± 41,6	153,3 ± 46,4	207,9 ± 57,8
**iScore 1 year**		100,6 ± 28,4	126,0 ± 32,2	169,8 ± 41,8
		**Count (Row N %)**	**Count (Row N %)**	**Count (Row N %)**
**Sex**	**Female**	29 (10,5)	193 (69,9)	54 (19,6)
	**Male**	8 (3,1)	157 (60,9)	93 (36,0)
**CNS 0**	**No**	37 (7,2)	345 (67,1)	132 (25,7)
	**Yes**	0 (0,0)	5 (25,0)	15 (75,0)
**CNS =<4**	**No**	36 (7,8)	314 (68,4)	109 (23,7)
	**Yes**	1 (1,3)	36 (48,0)	38 (50,7)
**CNS 5-7**	**No**	26 (7,9)	231 (70,0)	73 (22,1)
	**Yes**	11 (5,4)	119 (58,3)	74 (36,3)
**CNS >=8**	**No**	12 (4,0)	160 (53,5)	127 (42,5)
	**Yes**	25 (10,6)	190 (80,9)	20 (8,5)
**Stroke subtype**	**Lacunar origin**	24 (9,8)	198 (81,1)	22 (9,0)
	**Nonlacunar origin**	12 (5,5)	131 (59,8)	76 (34,7)
	**Underdetermined origin**	1 (1,4)	21 (29,6)	49 (69,0)
**Risk factor**	**Atrial fibrillation**	16 (20,0)	58 (72,5)	6 (7,5)
	**CHF**	7 (10,4)	54 (80,6)	6 (9,0)
	**Previous MI**	8 (5,4)	127 (85,8)	13 (8,8)
	**Current smoker**	4 (4,0)	64 (64,0)	32 (32,0)
**Current smoker**	**No**	30 (6,9)	292 (67,3)	112 (25,8)
	**Yes**	7 (7,0)	58 (58,0)	35 (35,0)
**Cancer**	**No**	36 (7,5)	334 (69,4)	111 (23,1)
	**Yes**	1 (1,9)	16 (30,2)	36 (67,9)
**Renal dialysis**	**No**	37 (7,0)	349 (66,5)	139 (26,5)
	**Yes**	0 (0,0)	1 (11,1)	8 (88,9)
**Dependent**	**No**	36 (8,3)	314 (72,2)	85 (19,5)
	**Yes**	1 (1,0)	36 (36,4)	62 (62,6)
**Glucose on admission above 135**	**No**	25 (8,5)	218 (73,9)	52 (17,6)
	**Yes**	12 (5,0)	132 (55,2)	95 (39,7)
**Mortality 30 days**	**No**	37 (8,1)	338 (74,0)	82 (17,9)
	**Yes**	0 (0,0)	12 (15,6)	65 (84,4)
**Mortality 1 year**	**No**	34 (8,7)	311 (79,1)	48 (12,2)
	**Yes**	3 (2,1)	39 (27,7)	99 (70,2)

SD indicates standard deviation; CHF: congestive heart failure; MI: myocardial infarction; CNS: Canadian neurological scale.

**Figure 1:**
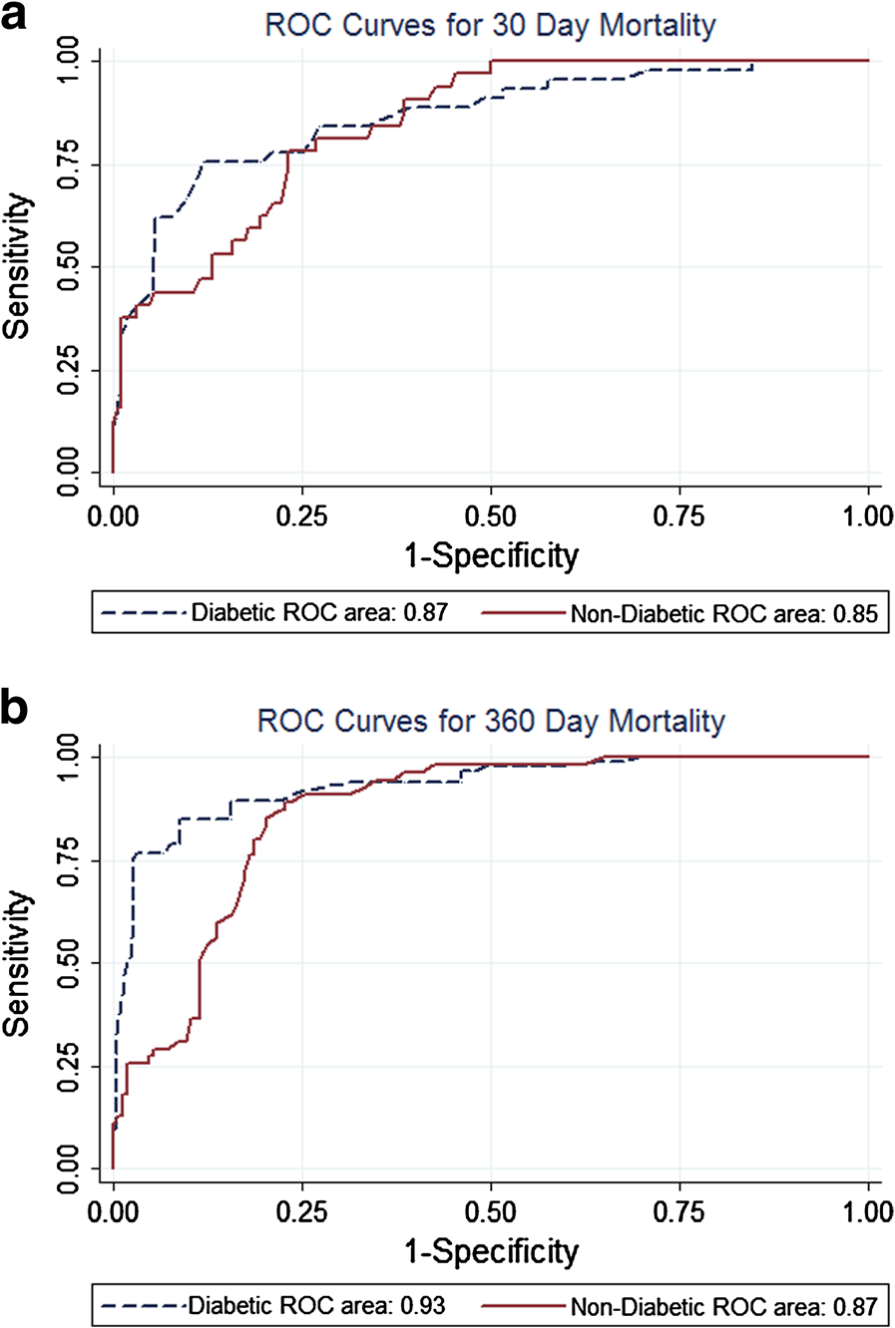
**ROC curves for IScore regarding a) 30-day and b) 1-year actual mortality rates.** Receiver operating characteristic (ROC) curves for IScore regarding 30-day and 1-year actual mortality rates for acute ischemic stroke diabetic (n = 312) and non diabetic patients (n = 222). The area under the ROC curve was 0.87 (95% CI, 0.80-0.93) for diabetic and 0.85 (95% CI, 0.79-0.91) for non-diabetic at 30 days and 0.93 (95% CI, 0.90-0.97) and 0.87 (95% CI, 0.82-0.91) at 1 year.

Discrimination, or predictive accuracy, was assessed by building receiver operating characteristic (ROC) curves for mortality both in diabetic and non-diabetic population for 30-day and 1 year mortality and the area under the curves (AUC) was used to evaluate and compare the predictive accuracy of risk classifications. Based on AUC analysis the discriminative ability of IScore was excellent for patients with diabetes with a value of 0.87 (95% CI, 0.80-0.93) and patients without diabetes with a value of 0.85 (95% CI, 0.79-0.91) at 30 days without significant difference between groups (Chi = 0.17, p = 0.68) (Figure 1). Respectively discriminative ability was also excellent for diabetic group with a value of 0.93 (95% CI, 0.90-0.97) and non-diabetic group with a value of 0.87 (95% CI, 0.82-0.91) at 1 year with significantly higher discriminative ability (Chi = 5.23, p < 0.05) for IS patients with diabetes especially located in high risk (IScore >160) diabetic subgroups (Figure 1).

In order to evaluate the model’s validity on the basis of its calibration, which represents the precision of the probabilities generated by a prediction model, we tabulated IScore categories and mortality in contingency tables. Using Hosmer–Lemeshow goodness-of-fit test, which is the most commonly used statistic method for contingency tables [18], we compared the estimated predicted mortality from the logistic regression models with the observed outcomes, for each risk category, of IScore prediction model. The Hosmer-Lemeshow test showed that the model’s calibration was satisfactory both in diabetic (p = 0.18) and non-diabetic population (p = 0.19).

## Discussion

Prognosis prediction after stroke is a field of intense clinical research and several prognostic models have been developed [19-22] towards this direction and among them IScore has been proposed as a well validated tool for mortality prediction [11,12]. Recently, IScore has also been reported to reliably predict short term functional outcome and clinical response to thrombolytic therapy [23,24] after acute IS as well as risk of hemorrhagic complications after thrombolytic therapy [24] and the above are especially important in the case of IS patients with diabetes given the lower rates of favorable outcomes after thrombolytic therapy observed in this group of stroke patients [25].

The development and broad application of a predictive tool for stroke mortality and functional recovery is of great importance today since it could identify patients at increased risk, guide level of inpatient and outpatient care and help neutralize health care disparities. Stroke is considered a continuum today commencing before admission and continuing after discharge. Addressing risk factors, improving quality of life and determining targeted interventions and an evidence-based discharge plan are considered fundamental factors determining outcome and reducing re-hospitalization rates and morbidity and mortality [26,27].

In the present study we investigated the validity of IScore in the prediction of 30-day and 1-year mortality in IS patients with and without diabetes and our results clearly indicate that IScore accurately predicts mortality in both groups of IS patients. Additionally, according to our findings, IScore’s predictive ability is higher in IS patients with diabetes for 1 year mortality and this superiority is specifically located at high risk subgroups.

Diabetes has been associated with increased risk of stroke at a younger age and, not uniformly, with increased stroke severity and mortality [4,8-10] as well as poorer favorable outcomes after thrombolytic therapy [25] and in our cohort patients with diabetes presented more frequently with lacunar strokes and with more severe scores in the Canadian Neurological Scale and higher IScores. Our results regarding increased incidence of lacunar strokes in patients with diabetes are in accordance with the findings of the majority of studies [4,28-32] although there is not uniformity of findings in all studies in this field [7].

Mortality rates after IS were similar for patients with and without diabetes at 30 days and 1 year post stroke in our study and the above are in accordance with the findings of Camalesh *et al* who also reported similar stroke mortality for patients with and without diabetes at 60 days and 1 year after IS [33] and Megherbi *et al* who reported similar post stroke mortality IS patients with and without diabetes at 3 months after IS [4]. Many studies have reported increased stroke mortality in patients with diabetes at different time points after ischemic stroke [8,9,34-38] with the majority of studies with long term follow up to report increased mortality. From the above it can be inferred that diabetes seems to increase stroke mortality on the long term while short term post stroke mortality may be similar between IS patients with diabetes and without diabetes and this is also in accordance with the slower post stroke recovery and increased post stroke disability reported for patients with diabetes [8,9].

This is the first study to evaluate IScore’s validity in another population sample, outside Canada, and to specifically examine validity in IS patients with diabetes. Our study was also conducted in a merely Caucasian population. Ethnicity has not been included as a parameter in the initial publication of IScore presentation due to lack of data for all patients although it was conferred from the study loci that it has been conducted in a mainly Caucasian population [11].

Race and ethnicity influence both incidence and prognosis of stroke with African Americans, Hispanic Americans and American Indians/Alaska natives to be at increased risk [39] while stroke mortality varies with African Americans to consistently exhibit higher stroke mortality rates than Caucasians [39,40] and this is attributed to increased stroke severity, lower socioeconomic status, variations in risk factors (uncontrolled blood pressure, smoking, inactivity, diabetes), general lower life expectancy and lack of access to medical care [39-41]. From the above point of view evaluation of IScore’s validity in other racial groups is of great interest since it could unveil any peculiarities or differences although it is expected to exert high validity. Especially for African Americans, given the higher prevalence of risk factors and greater stroke severity, IScore is expected, from the theoretical point of view and according to our findings, to exhibit even higher than the hereby reported validity.

The main weakness of the present study has been the relatively small, although sufficient, population sample (312 IS patients with diabetes and 222 without diabetes). A larger population sample could have allowed us to achieve even greater statistical power and this was more obvious in the case of comorbid patient conditions where we chose to report the results of Fisher’s exact test since the expected values were relatively small.

Our results further support the broad use of IScore as a tool for mortality prediction in IS stroke patients. IScore’s validity in mortality prediction in IS patients with diabetes is considered important given the explosive increase in the incidence of type II diabetes [42], its close correlation with stroke [43-45] as well as the increased stroke severity, poorer prognosis and response to thrompolytic therapy in IS patients with diabetes [25].

## Conclusions

In conclusion, IScore exhibits excellent predictive accuracy and good calibration in predicting 30 day and 1 year mortality in type 2 diabetic and non-diabetic IS Caucasian patients with even higher accuracy in predicting 1 year post-stroke mortality in patients with type 2 diabetes especially in high risk patients.

## Abbreviations

IS: Ischemic stroke; CT: Computed tomography; TOAST: Trial of ORG 10172 in acute stroke treatment; CAD: Coronary artery disease; CHF: Congestive heart failure; ROC curves: Receiver operating characteristic curves; AUC: Area under the curves; CNS: Canadian neurological scale; MI: Myocardial infarction.

## Competing interests

The authors declare that they have no competing interests.

## Authors’ contributions

VD carried out part of the research, helped to draft the manuscript, and performed the initial statistical report. KNT carried out the results evaluation, helped to draft the manuscript, and participated in the design and coordination of the study. GIP carried out part of the research, helped to draft the manuscript, and participated in the design and coordination of the study. KK performed the statistical analysis. EF carried out part of the research. MV carried out part of the research. GE carried out part of the research. NM carried out part of the research, and performed the data collection. KTK carried out part of the research. AM conceived the protocol design, and consulted to draft the manuscript. All authors read and approved the final manuscript.

## Pre-publication history

The pre-publication history for this paper can be accessed here:

http://www.biomedcentral.com/1471-2377/13/121/prepub

## References

[B1] Langhorne P Coupar F Pollock A Motor recovery after stroke: a systematic review Lancet Neurol 2009 8 8 741 754 10.1016/S1474-4422(09)70150-4 19608100

[B2] Kinlay S Changes in stroke epidemiology, prevention and treatment Circulation 2011 124 19 e494 e496 10.1161/CIRCULATIONAHA.111.069633 22064961 PMC4501778

[B3] Kissela BM Khoury J Kleindorfer D Woo D Schneider A Alwell K Miller R Ewing I Moomaw CJ Szaflarski JP Gebel J Shukla P Broderick JP Epidemiology of ischemic stroke in patients with diabetes: the greater Cincinnati/Northern Kentucky stroke study Diabetes Care 2005 28 2 355 359 10.2337/diacare.28.2.355 15677792

[B4] Megherbi SE Milan C Minier D Courveur G Osseby GV Tilling K Di Carlo A Inzitari D Wolfe CD Moreau T Giroud M Association between diabetes and stroke subtype on survival and functional outcome 3 months after stroke: data from the European BIOMED stroke project Stroke 2003 34 3 688 694 10.1161/01.STR.0000057975.15221.40 12624292

[B5] Kothari V Stevens RJ Adler AI Stratton IM Manley SE Neil HA Holman RR UKPDS 60: risk of stroke in type 2 diabetes estimated by the UK prospective diabetes study risk engine Stroke 2002 33 7 1776 1781 10.1161/01.STR.0000020091.07144.C7 12105351

[B6] Harmsen P Lappas G Rosengren A Wilhelmsen L Long-term risk factors for stroke: twenty-eight years of follow-up of 7457 middle-aged men in Goteborg, Sweden Stroke 2006 37 7 1663 1667 10.1161/01.STR.0000226604.10877.fc 16728686

[B7] Janghorbani M Hu FB Willett WC Li TY Manson JE Logroscino G Rexrode KM Prospective study of type 1 and type 2 diabetes and risk of stroke subtypes: the nurses’ health study Diabetes Care 2007 30 7 1730 1735 10.2337/dc06-2363 17389335

[B8] Sprafka JM Virnig BA Shahar E McGovern PG Trends in diabetes prevalence among stroke patients and the effect of diabetes on stroke survival: the Minnesota heart survey Diabet Med 1994 11 7 678 684 10.1111/j.1464-5491.1994.tb00332.x 7955994

[B9] Jorgensen H Nakayama H Raaschou HO Olsen TS Stroke in patients with diabetes. The Copenhagen stroke study Stroke 1994 25 10 1977 1984 10.1161/01.STR.25.10.1977 8091441

[B10] De Silva DA Ebinger M Christensen S Parsons MW Levi C Butcher K Barber PA Bladin C Donnan GA Davis SM Baseline diabetic status and admission blood glucose were poor prognostic factors in the EPITHET trial Cerebrovasc Dis 2010 29 1 14 21 10.1159/000255969 19893307

[B11] Saposnik G Kapral MK Liu Y Hall R O'Donnell M Raptis S Tu JV Mamdani M Austin PC IScore: A risk score to predict death early after hospitalization for an acute ischemic stroke Circulation 2011 123 7 739 749 10.1161/CIRCULATIONAHA.110.983353 21300951

[B12] Bushnell C Another score to predict ischemic stroke mortality? Circulation 2011 123 7 712 713 10.1161/CIRCULATIONAHA.110.012534 21300950

[B13] Recommendations on stroke prevention, diagnosis and therapy. Report of the WHO task force on stroke and other cerebrovascular disorders Stroke 1989 20 10 1407 1431 2799873 10.1161/01.str.20.10.1407

[B14] Adams HP Jr Bendixen BH Kappelle LJ Biller J Love BB Gordon DL Marsh EE 3rd Classification of subtype of acute ischemic stroke. Definitions for use in a multicenter clinical trial. TOAST. Trial of Org 10172 in acute stroke treatment Stroke 1993 24 1 35 41 10.1161/01.STR.24.1.35 7678184

[B15] Hanley JA McNeil BJ The meaning and use of the area under a receiver operating characteristic (ROC) curve Radiology 1982 143 1 29 36 7063747 10.1148/radiology.143.1.7063747

[B16] Swets JA Measuring the accuracy of diagnostic systems Science 1988 240 4857 1285 1293 10.1126/science.3287615 3287615

[B17] Hosmer DW Taber S Lemeshow S The importance of assessing the fit of logistic regression models: a case study Am J Publ Health 1991 81 12 1630 1635 10.2105/AJPH.81.12.1630 PMC1405276 1746660

[B18] Hosmer DW Lemeshow S Assessing the fit of the model, applied logistic regression 1989 New York: Wiley 135 175

[B19] Solberg OG Dahl M Mowinckel P Stavem K Derivation and validation of a simple risk score for predicting 1-year mortality in stroke J Neurol 2007 254 10 1376 1383 10.1007/s00415-007-0555-2 17934885

[B20] Johnston KC Connors AF Jr Wagner DP Haley EC Jr Predicting outcome in ischemic stroke: external validation of predictive risk models Stroke 2003 34 1 200 202 10.1161/01.STR.0000047102.61863.E3 12511774 PMC2748724

[B21] Williams GR Jiang JG Development of an ischemic stroke survival score Stroke 2000 31 10 2414 2420 10.1161/01.STR.31.10.2414 11022073

[B22] Smith EE Shobha N Dai D Olson DM Reeves MJ Saver JL Hernadez AF Peterson ED Fonarow GC Schwamm LH Risk score for in-hospital ischemic stroke mortality derived and validated within the get with the guidelines-stroke program Circulation 2010 122 15 1496 1504 10.1161/CIRCULATIONAHA.109.932822 20876438

[B23] Saposnik G Raptis S Kapral MK Liu Y Tu JV Mamdani M Austin PC The iScore predicts poor functional outcomes early after hospitalization for an acute ischemic stroke Stroke 2011 42 12 3421 3428 10.1161/STROKEAHA.111.623116 21960583

[B24] Saposnik G Fang J Kapral MK Tu JV Mamdani M Austin P Johnston CS The iScore predicts effectiveness of thrombolytic therapy for acute ischemic stroke Stroke 2012 43 5 1315 1322 10.1161/STROKEAHA.111.646265 22308252

[B25] Nikneshan D Raptis R Pongmoragot J Zhou L Johnston SC Saposnik G Predicting clinical outcomes and response to thrombolysis in acute stroke patients with diabetes Diabetes Care 2013 36 7 2041 2047 10.2337/dc12-2095 23359359 PMC3687301

[B26] Saposnik G Kapral MK Poststroke care: chronicles of a neglected battle Stroke 2007 38 6 1727 1729 10.1161/STROKEAHA.107.487249 17510444

[B27] Birbeck GL Zingmond DS Cui X Vickrey BG Multispecialty stroke services in California hospitals are associated with reduced mortality Neurology 2006 66 10 1527 1532 10.1212/01.wnl.0000203993.93763.b8 16540604

[B28] Baliga BS Weinberger J Diabetes and stroke: part one–risk factors and pathophysiology Curr Cardiol Rep 2006 8 1 23 28 10.1007/s11886-006-0006-1 16507231

[B29] Licata G Tuttolomondo A Pinto A Association between diabetes and stroke subtype on survival and functional outcome 3 months after stroke: data from the European BIOMED stroke project Stroke 2004 35 3 e61 10.1161/01.STR.0000117968.13015.C4 14976331

[B30] Tuttolomondo A Pinto A Salemi G Di Raimondo D Di Sciacca R Fernandez P Ragonese P Savettieri G Licata G Diabetic and non-diabetic subjects with ischemic stroke: differences, subtype distribution and outcome Nutr Metab Cardiovasc Dis 2008 18 2 152 157 10.1016/j.numecd.2007.02.003 17702553

[B31] Ohira T Shahar E Chambless LE Rosamond WD Mosley TH Folsom AR Risk factors for ischemic stroke subtypes: the atherosclerosis risk in communities study Stroke 2006 37 10 2493 2498 10.1161/01.STR.0000239694.19359.88 16931783

[B32] Arboix A Rivas A Garcia-Eroles L De Marcos L Massons J Oliveres M Cerebral infarction in diabetes: clinical pattern, stroke subtypes, and predictors of in-hospital mortality BMC Neurol 2005 5 1 9 10.1186/1471-2377-5-9 15833108 PMC1097737

[B33] Kamalesh M Shen J Eckert GJ Long term post-ischemic stroke mortality in diabetes: a veteran cohort analysis Stroke 2008 39 10 2727 2731 10.1161/STROKEAHA.108.517441 18658031

[B34] Laing SP Swerdlow AJ Carpenter LM Slater SD Burden AC Botha JL Morris AD Waugh NR Gatting W Gale EA Patterson CC Qiao Z Keen H Mortality from cerebrovascular disease in a cohort of 23000 patients with insulin-treated diabetes Stroke 2003 34 2 418 421 10.1161/01.STR.0000053843.03997.35 12574553

[B35] Olsson T Viitanen M Asplund K Eriksson S Hagg E Prognosis after stroke in diabetic patients. A controlled prospective study Diabetologia 1990 33 4 244 249 10.1007/BF00404803 2347437

[B36] Oppenheimer SM Hoffbrand BI Oswald GA Yudkin JS Diabetes mellitus and early mortality from stroke Br Med J 1985 291 6501 1014 1015 3931771 10.1136/bmj.291.6501.1014-a PMC1416950

[B37] Oliveira TV Gorz AM Bittencourt PR Diabetes mellitus as a prognostic factor in ischemic cerebrovascular diseases Arq Neuropsiquiatr 1988 46 3 287 291 3223832 10.1590/s0004-282x1988000300009

[B38] Eriksson M Carlberg B Eliasson M The disparity in long-term survival after a first stroke in patients with and without diabetes persists: the Northern Sweden MONICA study Cerebrovasc Dis 2012 34 2 153 160 10.1159/000339763 22907276

[B39] Roger VL Go AS Lloyd-Jones DM Benjamin EJ Berry JD Borden WB Bravata DM Dai S Ford ES Fox CS Fullerton HJ Gillespie C Hailpern SM Heit JA Howard VJ Kissela BM Kittner SJ Lackland DT Lichtman JH Lisabeth LD Makuc DM Marcus GM Marelli A Matchar DB Moy CS Mozaffarian D Mussolino ME Nichol G Paynter NP Soliman EZ Heart disease and stroke statistics – 2012 update: a report from the American heart association Circulation 2012 125 1 e2 e220 22179539 10.1161/CIR.0b013e31823ac046 PMC4440543

[B40] Gillum RF Stroke mortality in blacks: disturbing trends Stroke 1999 30 8 1711 1715 10.1161/01.STR.30.8.1711 10436126

[B41] Cruz-Flores S Rabinstein A Biller J Elkind MS Griffith P Gorelick PB Howard G Leira EC Morgenstern LB Ovbiagele B Peterson E Rosamond W Trimble B Valderrama AL Racial-ethnic disparities in stroke care: the American experience: a statement for healthcare professionals from the American heart association/American stroke Association Stroke 2011 42 7 2091 2116 10.1161/STR.0b013e3182213e24 21617147

[B42] King H Aubert RE Herman WH Global burden of diabetes, 1995–2025: prevalence, numerical estimates and projections Diabetes Care 1998 21 9 1414 1431 10.2337/diacare.21.9.1414 9727886

[B43] Towfighi A Markovic D Ovbiagele B Current national patterns of comorbid diabetes among acute ischemic stroke patients Cerebrovasc Dis 2012 33 5 411 418 10.1159/000334192 22456491

[B44] Venketasubramanian N Rother J Bhatt DL Pasquet B Mas JL Alberts MJ Hill MD Aichner F Steg PG Two-year vascular event rates in patients with symptomatic cerebrovascular disease: the REACH registry Cerebrovasc Dis 2011 32 3 254 260 10.1159/000328650 21876353

[B45] Lees KR Walters MR Acute stroke and diabetes Cerebrovasc Dis 2005 20 Suppl 1 9 14 16276080 10.1159/000088232

